# The rising burden of invasive fungal infections in COVID-19, can structured CT thorax change the game

**DOI:** 10.1186/s43055-022-00694-3

**Published:** 2022-01-06

**Authors:** Roopak Dubey, Kamal Kumar Sen, Sudhansu Sekhar Mohanty, Sangram Panda, Mayank Goyal, Sreedhar Mohan Menon

**Affiliations:** Department of Radio-Diagnosis, Kalinga Institute of Medical Sciences, KIIT Road, Patia, , Bhubaneswar, Odisha 751024 India

**Keywords:** Pulmonary fungal infections, Mucormycosis, Invasive candidiasis

## Abstract

**Background:**

The occurrence of invasive fungal infections in COVID-19 patients is on surge in countries like India. Several reports related to rhino-nasal-sinus mucormycosis in COVID patients have been published in recent times; however, very less has been reported about invasive pulmonary fungal infections caused mainly by mucor, aspergillus or invasive candida species. We aimed to present 6 sputum culture proved cases of invasive pulmonary fungal infection (four mucormycosis and two invasive candidiasis) in COVID patients, the clues for the diagnosis of fungal invasion as well as difficulties in diagnosing it due to superimposed COVID imaging features.

**Case presentation:**

The HRCT imaging features of the all 6 patients showed signs of fungal invasion in the form of cavities formation in the pre-existing reverse halo lesions or development of new irregular margined soft tissue attenuating growth within the pre-existing or in newly formed cavities. Five out of six patients were diabetics. Cavities in cases 1, 2, 3 and 4 of mucormycosis were aggressive and relatively larger and showed relatively faster progression into cavities in comparison with cases 5 and 6 of invasive candidiasis.

**Conclusion:**

In poorly managed diabetics or with other immunosuppressed conditions, invasive fungal infection (mucormycosis, invasive aspergillosis and invasive candidiasis) should be considered in the differential diagnosis of cavitary lung lesions.

## Background

The victims of COVID-19 are incredibly prone to fungal and bacterial infections, especially those in an intensive care unit (ICU). Aspergillosis, invasive candidiasis and mucormycosis are the most common associates [1–6]. The occurrence of these fungal co-infections is rising [1, 3, 4, 7, 8]. The disease is usually associated with comorbidities like haematologic malignancy, diabetes and impaired immunological systems [9]. Mucormycosis frequently affects the sinus, brain and respiratory system. Lung involvement with mucormycosis often deprived of an early specific treatment because of delay in diagnosis. High-resolution computed tomography (HRCT) of thorax plays an important role in detecting the varied features of this dreadful condition early, if a follow-up scan for COVID-19 patients is undertaken in about ten days or as per protocol or HRCT undertaken in patients with chest infection and breathlessness with pre-existing risk factors and co-morbidities during this pandemic situation.

We are presenting six cases of COVID-19, who developed invasive pulmonary fungal infection, four of them were mucor and remaining two were of candida species. We have also tried to set a protocol for undertaking HRCT chest, for early detection of any associated pulmonary fungal infection in COVID patients.

## Case presentation

### Case 1

A 55-year-old male patient presented with pyrexia, tachypnea, severe breathlessness and sore throat for 4 days. He was reverse-transcriptase polymerase chain reaction (RT-PCR) positive. He was a known diabetic being managed with oral hypoglycaemic drugs. Complete blood count revealed a haemoglobin level of 11 gm/dl, lymphopenia (10%; normal 20–40%). high C-reactive protein (CRP)—29.53 mg/l, high procalcitonin—0.89 ng/ml, elevated D-dimer assay. HRCT scan of the chest was done on 2nd day of admission, showed diffuse ground-glass opacities (GGO) in both lungs with patchy consolidations and reverse halo sign. CT severity score of 22/25 (typical for COVID-19).

**Fig. 1:**
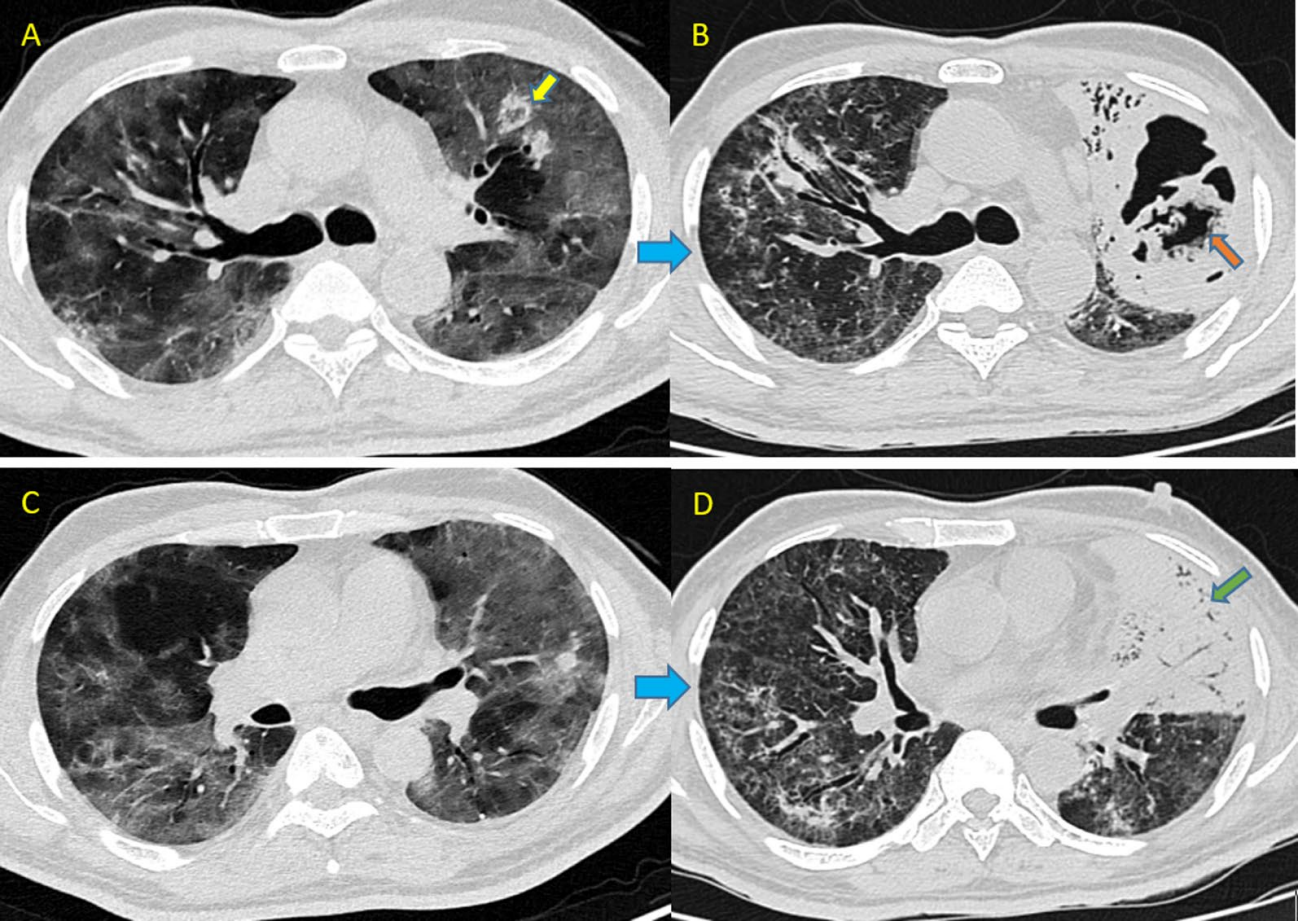
Imaging features of mucormycosis in Case 1 of COVID-19. **A**, **B** Axial HRCT scans show diffuse ground glass opacities in bilateral lung fields with few patches of reverse halo lesions (yellow arrow in **A**), without any evidence of cavitation. The scan was repeated after 21 days revealed cavitary lesions with soft tissue attenuating irregularly margined growth suggesting fungal infestation (orange arrow in **B**). **C**, **D** Revealed rapid progression of GGOs into consolidation (Green arrow in **D**)

During the course of his hospital stay, he was treated with intravenous antibiotics, steroids, anti-viral and multivitamins, as well as general supportive care. To avoid thrombotic problems, he was also given subcutaneous enoxaparin (40 mg/0.4 ml) twice a day. His diabetes was controlled with insulin doses adjusted on a sliding scale. Despite receiving the required therapy, his health worsened over the next few days. This necessitated the need for a repeat CT scan. Which was done on 21st day. Scan revealed diffuse GGOs along with interlobular septal thickening in all lobes and a large thick walled cavitary lesion surrounded by consolidation in left upper lobe. Curvilinear soft tissue density growth within irregular margins was noted within the cavity, indicating a high possibility of fungal infection (Fig. 1).

Sputum samples were then collected and sent for fungal culture which was positive for mucormycosis. Antibiotic susceptibility showed sensitivity to amphotericin B and voriconazole, while fluconazole showed intermediate sensitivity. Culture revealed insensitivity to caspofungin.

Another repeat scan was done on 30th day in order to track the progression of fungal infestation, showing no obvious change in characteristic of left upper lobar lesion. Patient became RTPCR negative on 32nd day and was shifted to high dependency unit (HDU) for further management. At the time of writing, he was on aggressive antifungals and antibiotics.

### Case 2

**Fig. 2:**
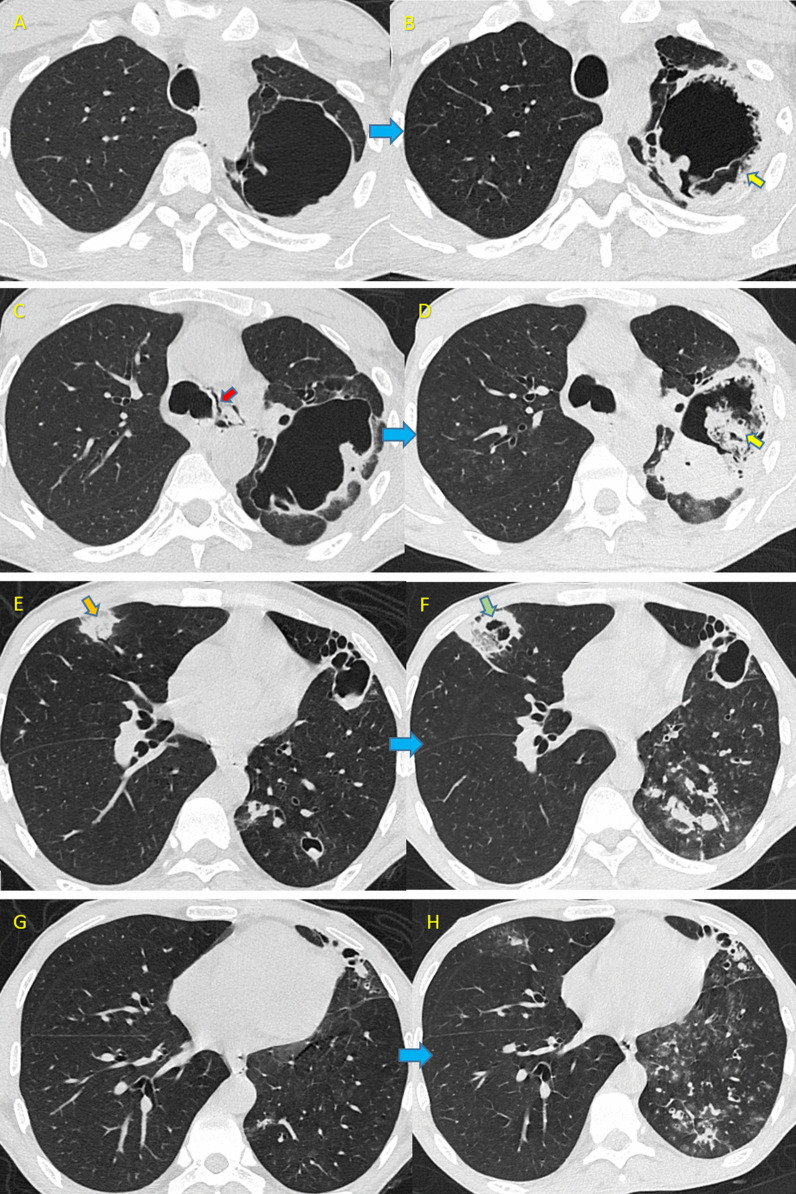
Axial HRCT scans showing imaging features of mucormycosis in case 2 of COVID-19. **A**–**D** CT axial images A and C show multiple cavitary lesions in the left upper and lower lobes. The repeat scans B and D show soft tissue attenuating growth (yellow arrows in **B** and **D**). Mild pneumomediastinum (red arrow in **C**) also noted. **E**, **F** A new cavity formed in the right upper lobe (green arrow in **F**) in pre-existing reverse halo sign (orange arrow in **E**). **G**, **H** Multiple soft tissue nodular opacities developed especially in left lower lobe in **H** in comparison with **G**

A 32-year-old male was admitted with fever and cough for 6 days. He had completed anti-tubercular treatment (ATT) in the past and was a known diabetic (5 years) with chronic kidney disease (CKD) on medical management. D-Dimer and CRP were elevated. The patient was managed conservatively with oxygen, intravenous antibiotics, injection dexamethasone and injection enoxaparin. CT chest done on the 3rd day of admission, revealed fibro-cavitary lesions, bilateral patchy consolidation with traction bronchiectatic changes in left lung. Some of the cavities showed fluid level. Fibro-cavitary lesion and ill-defined ground glass opacities were seen in left lower lobe. Pneumo-mediastinum was also noted (Fig. 2). In next few days, the oxygen requirement gradually increased and he developed severe respiratory distress with repeated desaturation for which he was admitted in ICU for respiratory support. Another CT scan was undertaken on the 9th day which revealed irregularly margined soft tissue attenuating structures, developing within the pre-existing cavities and some with fluid levels suggesting a fresh growth (Fig. 2). Culture for fungal infection was advocated on the basis of CT report. The sputum culture revealed mucormycosis for which amphotericin B was started. However, despite best efforts the patient succumbed to death on the 14th day.

### Case 3

**Fig. 3:**
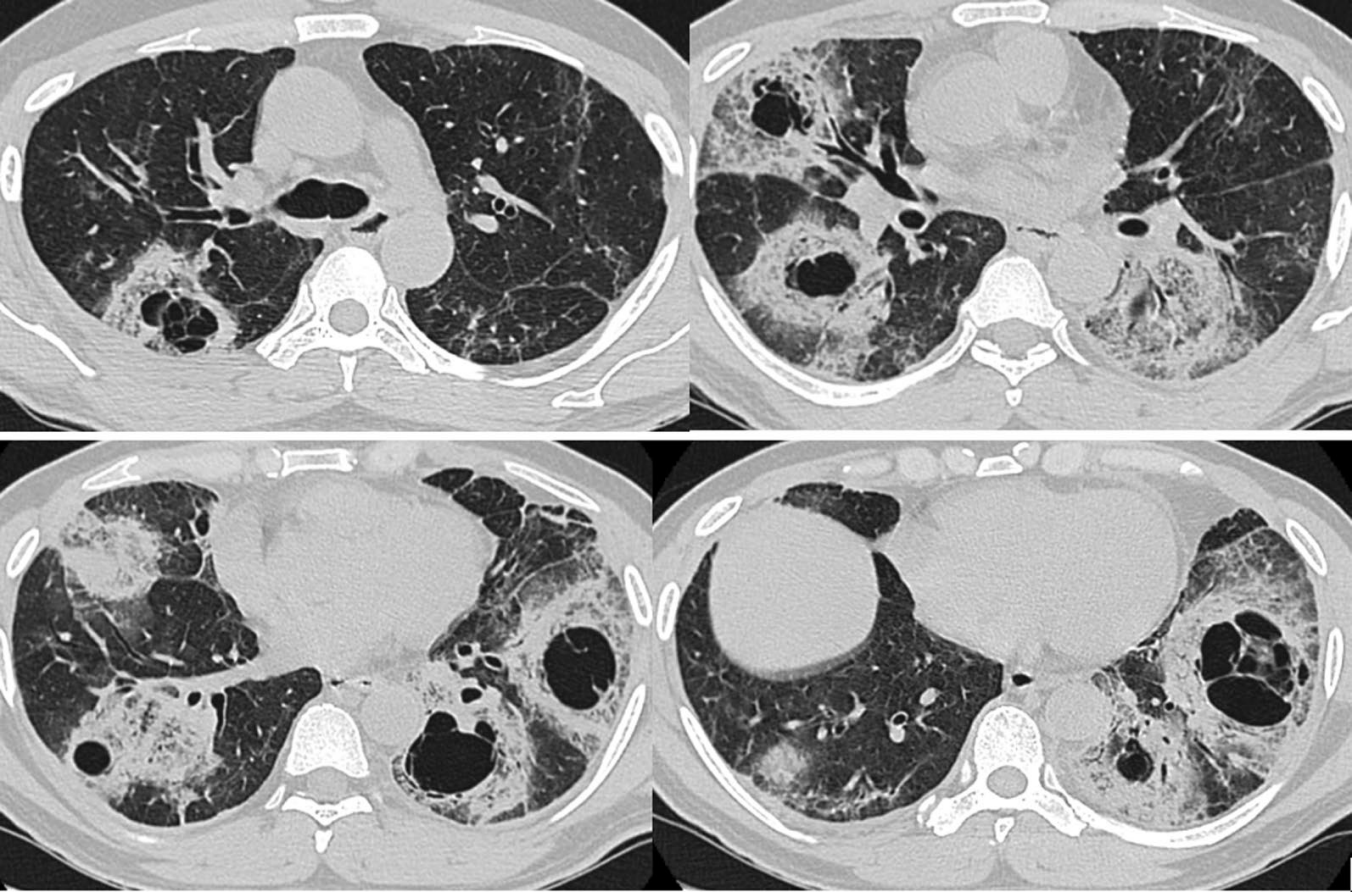
Axial HRCT scans showing imaging features of Mucormycosis in Case 3 of COVID-19. CT axial images shows multiple cavitary lesions with irregular nodular growth and internal septations, in both lungs, look like arising from pre-existing reverse halo sign

A 55-year-old COVID recovered patient came to our hospital after 15 days of discharge from another hospital. RTPCR for COVID virus came negative at the time of admission. He was a known diabetic patient and showing worsening of symptoms (breathlessness and cough) since last few days. His CRP was deranged and CBC revealed lymphopenia. HRCT thorax revealed multiple large cavitary lesions surrounded by ground glass opacities and consolidation in both lungs (Fig. 3). Thin internal septations were noted in the cavities. Initial baseline CT scan of patient was unavailable. Sputum culture of patient revealed mucor growth. At the time of writing, he was in critical condition and on antifungals and antibiotics.

### Case 4

**Fig. 4:**
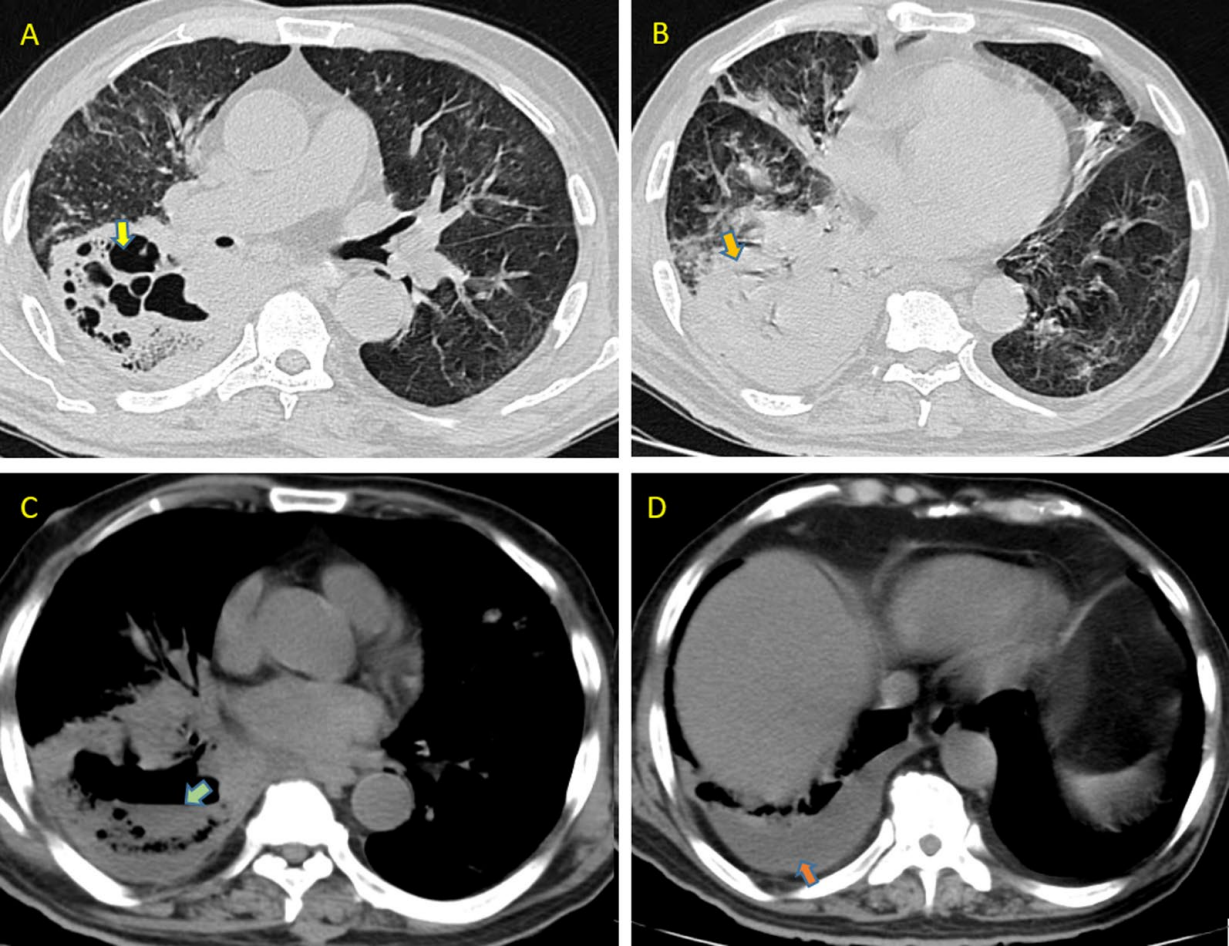
Axial HRCT scans showing imaging features of Mucormycosis in Case 4 of COVID-19. 4 **A**, **B** CT axial image large cavitary lesions (yellow arrow in **A**) with thick internal septations, surrounded by thick consolidation. Dense consolidation also noted in left lower lobe (orange arrow in **B**). **C** Air fluid level within cavity (green arrow in **C**). **D** Minimal right pleural effusion (dark orange arrow in **D**)

A 74-year-old elderly male admitted to our hospital after recovering from COVID-19. He was a known diabetic since 20 years. As the per the available information, he took the treatment from some other hospital including 8 mg injections of steroids twice a day. Initial baseline scan was unavailable. HRCT thorax done in our hospital revealed large thick walled cavitary lesion with internal septations and fluid level in the right lower lobe, suspicious of invasive fungal aetiology. Patchy areas of ill-defined ground glass opacities were also noted in both lungs along with minimal right pleural effusion (Fig. 4). On the basis of CT report, sputum culture was done which revealed mucor species. At the time of writing, the patient was in ICU and on antifungals and antibiotics as per recommendation by antibiotic susceptibility test.

### Case 5

A 52-year-old RTPCR positive male patient was admitted with complaints of fever, burning micturition and multiple joint pain since two months. Patient was a non-diabetic and without any other comorbidities. He was discharged after 4 days, advised home quarantine with oral medications.

**Fig. 5:**
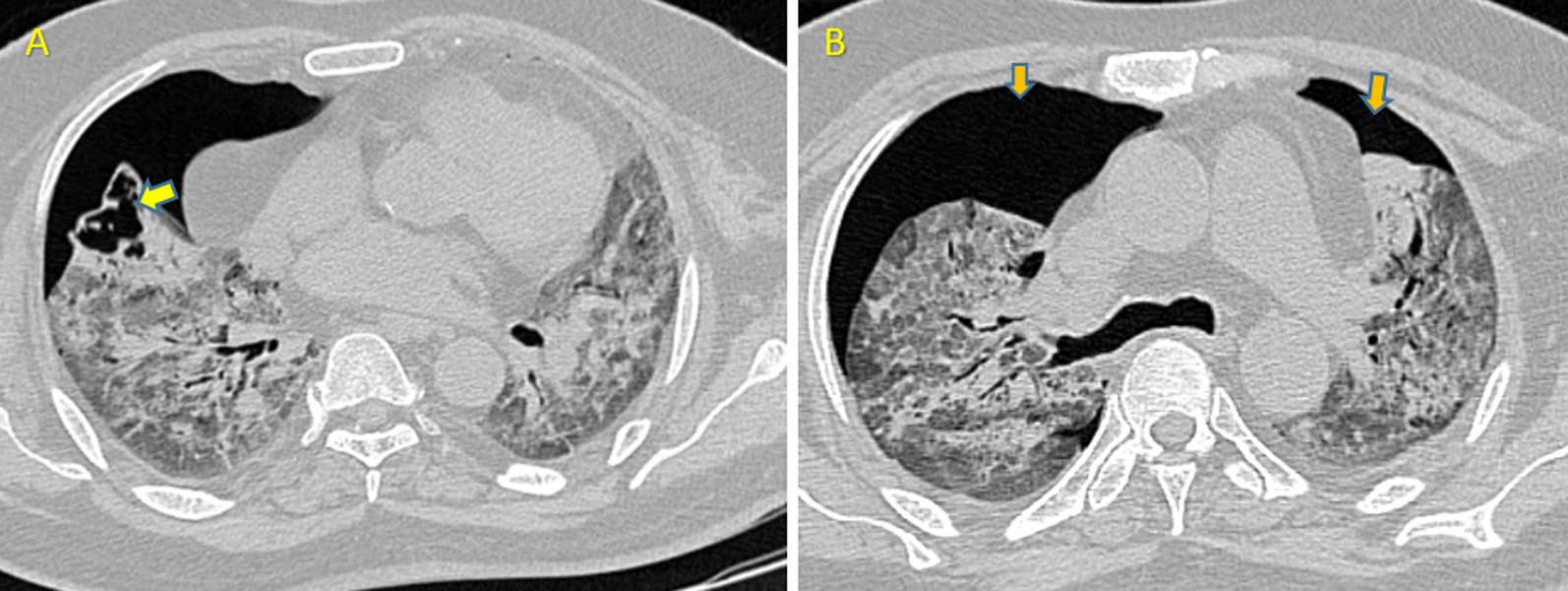
Axial HRCT scans showing imaging features of Candidiasis in case 5 of COVID-19. **A** Axial HRCT thorax of case 5 shows small cavitary lesions with few internal septations and tiny soft tissue nodular opacities within (yellow arrow in **A**). Sputum culture revealed Candida species. **B** Bilateral pneumothorax can be seen (orange arrows in **B**)

Patient was readmitted after 1 month with breathlessness, sore throat and cough. HRCT thorax revealed collapse and consolidation with diffuse GGOs in all lobes of bilateral lung fields. Bilateral pneumothorax (right > left) was also noted. Irregularly marginated cavitary lesion seen in right middle lobe with few internal septations pointing towards the possibility of fungal infection within the cavity (Fig. 5).

Sputum for acid fast bacilli was negative. Sputum for gram staining showed few epithelial cells, few gram-positive cocci and plenty of gram-positive budding yeast cells with pseudohyphae. Culture for the fungal infection revealed candida tropicalis with amphotericin B and fluconazole resistivity and caspofungin, flucytosine and micafungin sensitivity. Voriconazole showed intermediate sensitivity.

### Case 6

**Fig. 6:**
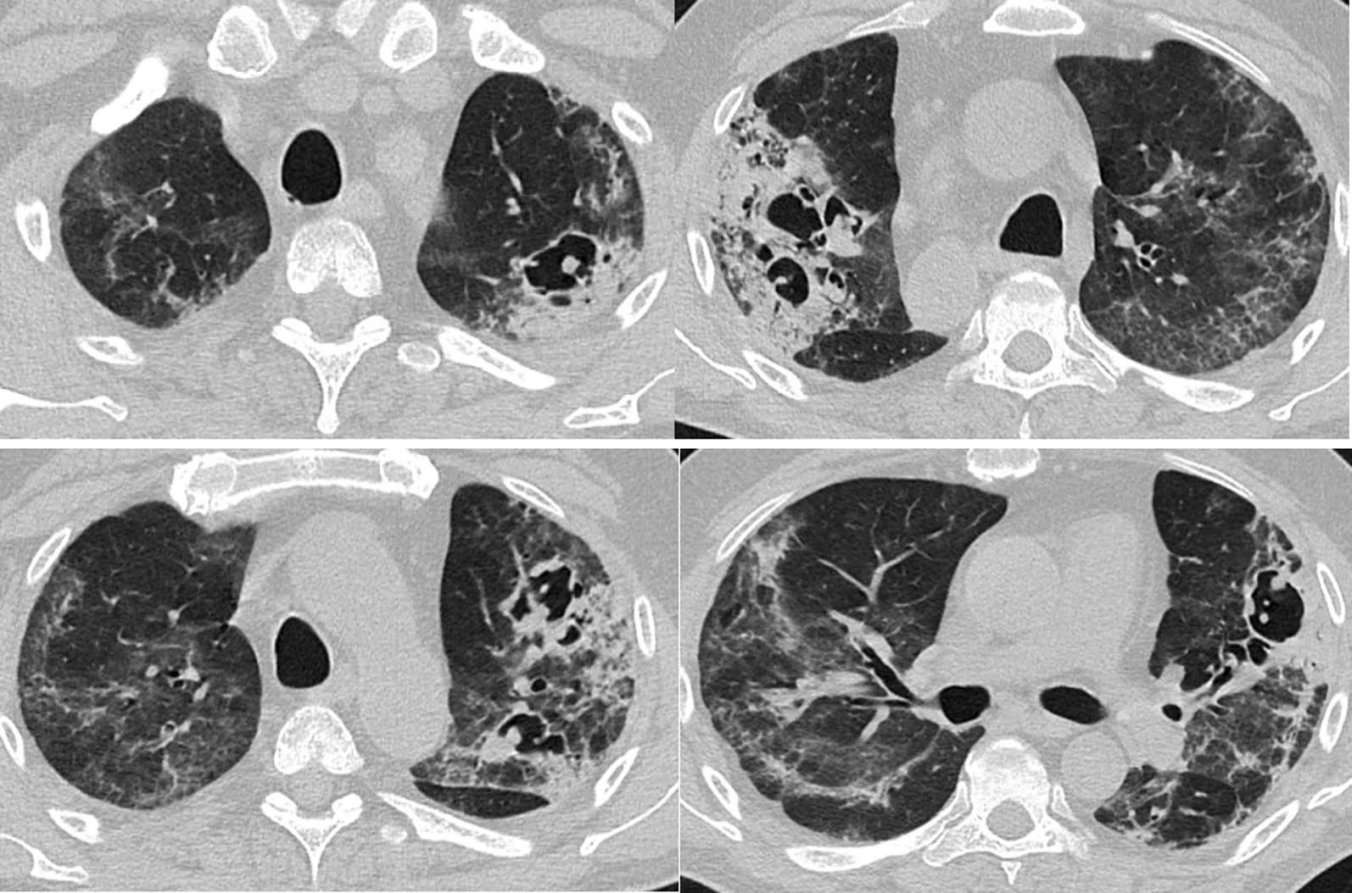
Axial HRCT images of case 6 shows multiple cavitary lesions in both lungs with irregularly margined soft tissue attenuation nodular growth. Sputum culture revealed Candida species

A 45-year-old man, RTPCR positive, was admitted with complaints of fever cough and breathlessness for 3 days. He was a known diabetic since 10 years. At the time of admission, CRP and D-dimer were raised significantly. He was managed with COVID protocol. HRCT thorax revealed patchy areas GGOs, consolidations with some areas of interlobular septal thickening seen bilaterally. Multiple areas of cavitary changes were seen in bilateral upper lobes and right middle lobe. Some of the cavities showed irregular soft tissue density structures within, suggesting a fungal growth (Fig. 6). Sputum culture grew candida albicans with amphotericin B, caspofungin, fluconazole, flucytosine, micafungin sensitivity and voriconazole resistivity.

## Discussion

As the COVID-19 pandemic continues, more specialists are becoming aware of fungal co-infections. Aspergillus, candida and mucor are the most common fungi detected so far. Most of the COVID-19 patients did not have a sputum fungal evaluation at the start of their treatment. Also, detecting fungus with a single sputum fungal culture is often difficult [10]. Thus, detection of fungal characteristics on HRCT thorax is a useful tool in this pandemic, because of increasing incidence of superimposed fungal infection.

Diabetes, glucocorticoids, hematopoietic malignancy, persistent neutropenia, hematopoietic stem cell transplantation and trauma are all associated with an increased risk of mucormycosis in COVID-19 patients [11]. In our series, 5 out of 6 patients were diabetics and all patients were on corticosteroid therapy due to COVID infection.

Imaging might be non-specific for the pulmonary mucormycosis. Early imaging may show peri-bronchial GGOs. Subsequently, the illness advances into consolidation or nodules with a CT halo sign, followed by central necrosis and the creation of cavities [12]. The presence of pleural effusion also favours mucor [13]. The reverse halo sign can help distinguish mucor from other fungal pneumonias (like aspergillus) [14]. Case 1 in our presentation, in period of 21 days or less, had developed a large cavitary lesion with soft tissue attenuating growth along the margins of the cavity and this cavitary lesion was formed in the region where two patches of small reverse halo lesions were present in the first CT scan. Because of rapid progression of the small reverse halo lesions into a large cavitation with surrounding consolidation within a short time (~ 21 days), we suspected it to be a fungal lesion. Rapid progression of GGOs into consolidation was also noted. Sputum culture confirmed mucor species. We inferred that the rapidly progressing cavity with soft tissue attenuating growth within, along with surrounding consolidation, was caused by superimposed mucor infection in this COVID patient.

In patient 2, two HRCT scans were done at an interval of 9 days. Initial scan showed cavitary lesions with few smooth internal septations within. Subsequent scan revealed new irregularly margined soft tissue attenuating growth along the walls within the cavity. A new cavity was seen forming in anterior segment of right upper lobe in the region of pre-existing reverse halo lesion. Mucor species grew on sputum culture. Patient was a known case of old pulmonary tuberculosis (PTB). Mucor can form cavities by itself and may show growth within, but in this case due to the absence of previous base line CT scan it was difficult to differentiate mucor from PTB. Even if the cavity was pre-existing due to PTB the new irregular soft tissue attenuating growth along the wall and within the cavity was definitely suggestive of fungal invasion. Moreover, newly formed cavity in pre-existing reverse halo sign favours fungal infestation.

Cases 3 and 4 were recovered COVID patients who were re-admitted due to worsening of symptoms. Both were diabetics. Baseline initial HRCT thorax was not available. CT axial images in case 3 showed multiple cavitary lesions with irregular nodular growth and internal septations, in both lungs, looks like arising from pre-existing reverse halo sign, favouring the fungal infection, came out to be mucor on sputum culture. Large thick walled cavitary lesion with internal septations and fluid level in case 4 strongly suggested fungal aetiology. Minimal right pleural effusion was favouring mucor proved on sputum culture. We infer that a repeat CT scan should be undertaken if already recovered COVID patients again comes with progression or resurgence of symptoms and the presence of cavities on CT should be followed by fungal related investigations.

In severe COVID-19 patients with a broader spectrum of antibacterial medications, parenteral diet and invasive examinations, or in patients with persistent neutropenia and other immune disability causes, the risk of Candida infection may increase dramatically [15]. In our 5th and 6th case, HRCT thorax revealed irregularly margined cavitary lesions within the lungs pointing towards the possibility of fungal infection. Sputum culture revealed Candida species in both of these patients.

After closely observing all the scans of these patients, we infer that it is very difficult to differentiate the superadded fungal infection in COVID patients as GGOs, consolidation and reverse halo signs are commonly seen in both fungal and COVID infections. It becomes more difficult when there is diffuse involvement of lungs in COVID (deviating from the normal peripheral involvement) usually seen in severe patients. However, some clues that can suggest pulmonary fungal infestation are the presence of cavities with soft tissue attenuating irregular growth, pleural effusion and unusual rapid conversion of reverse halo to cavities and consolidation. In patients 1 and 2, new cavities evolved in the region where there were reverse halo lesions in the previous scans; hence in the presence of reverse halo in severe COVID patients with pre-existing risk factors (diabetes and immunocompromised status), a repeat scan in 2–4 weeks or earlier should be the norm.

It is very difficult to differentiate between different invasive fungal species radiologically especially in COVID scenario, as all of them show overlapping radiological features among themselves and with COVID. However, presence of more than 10 nodules with pleural effusion and reverse halo sign is in favour of pulmonary mucormycosis rather than aspergillosis or other fungal infections [13]. Pleural effusion was seen in case 4 of mucormycosis and new cavities were formed in pre-existing reverse halo sign in cases 1, 2 and 3 of mucormycosis. The most common thin-section CT findings of pulmonary candidiasis are multiple bilateral nodular opacities often associated with areas of consolidation [16]. Multiple cavitary lesions with surrounding consolidation may also be seen in pulmonary candidiasis [17]. This was correlating with our findings in cases 5 and 6 of candidiasis. We found that although invasive candidiasis showed cavities, yet their sizes were small and progression was relatively slow. In our study, cases 1, 2, 3 and 4 showed relatively rapid progression of cavities that could be attributed to highly invasive nature of mucor species. In general, pulmonary mucormycosis is rare in comparison with pulmonary aspergillosis and candidiasis, but in this COVID crisis, there is surge in mucor infections due to rampant steroid use and high prevalence of diabetes in countries like India.

We suggest, in severe COVID patients (specially with diabetes and immunosuppressive states), close monitoring and follow-up for the cavitary lesions with CT scan should be undertaken. Appearance of new cavities or soft tissue attenuating growth within existing cavity should be reported as probable fungal infection in this pandemic until proved otherwise and empirical antifungals should be started as soon as possible because the superimposed fungal infection with COVID has significantly higher mortality. Once the diagnosis of pulmonary fungal infection is documented, other organs specially brain and paranasal sinuses should be examined clinically and radiologically as these regions are also commonly affected by fungal species. The most common fungal pathogens associated with CNS infections include candida and aspergillus species, and mucorales fungi [18].

There seems to be a variety of factors that might lead to fungal infections in these four COVID-19 patients: (1) mucormycosis is more likely to occur if diabetes is present. (2) Uncontrolled hyperglycaemia is frequently seen as a result of corticosteroid use. Acidosis causes a low pH, which is ideal for mucor spores to grow. (3) COVID-19 frequently causes endothelialitis, endothelial damage, lymphopenia, thrombosis and a decrease in CD4+ and CD8+ levels, putting the patient at risk for opportunistic fungal infection. (4) For mucormycosis, free iron is a great resource. Hyperglycaemia causes transferrin and ferritin to be glycosylated, which lowers iron binding and allows for more free iron. Furthermore, a rise in cytokines, particularly interleukin-6, increases free iron via raising ferritin levels due to increased synthesis and reduced iron transport in COVID-19 patients. (5) In the setting of diminished WBC phagocytic activity, mucor formation is encouraged by high glucose, low pH, and free iron [19].

Pneumomediastinum and pneumothorax in cases 2 and 5, respectively, were without any iatrogenic cause, leading us to infer that this was a COVID-related complication rather than a result of mechanical or barotrauma [20, 21] The widespread alveolar damage to serious COVID conditions might be one probable mechanism in this case in which alveoli are prone to rupture [21].

Since HRCT thorax has become one of the most widely used diagnostic investigations in COVID patients, identifying radiological characteristics of fungal infection in COVID can thus be a valuable for triaging. In all of our cases, the radiological findings on HRCT thorax prompted the clinicians to rule out fungal infections. It was felt that a HRCT on admission and discharge (10–14 days approximately) and a follow-up scan after 2 to 4 weeks (if the patient showed worsening of symptoms) for analysis by skilful radiologists will be beneficial in picking up early fungal attack, if any, to pick up the effects of this deadly virus early, to avoid treatment delays and enhance the chances of survival.

We could not include imaging features of pulmonary aspergillosis with COVID-19 patients as no case came till writing of this report. This is the limitation of this article.

**Fig. 7:**
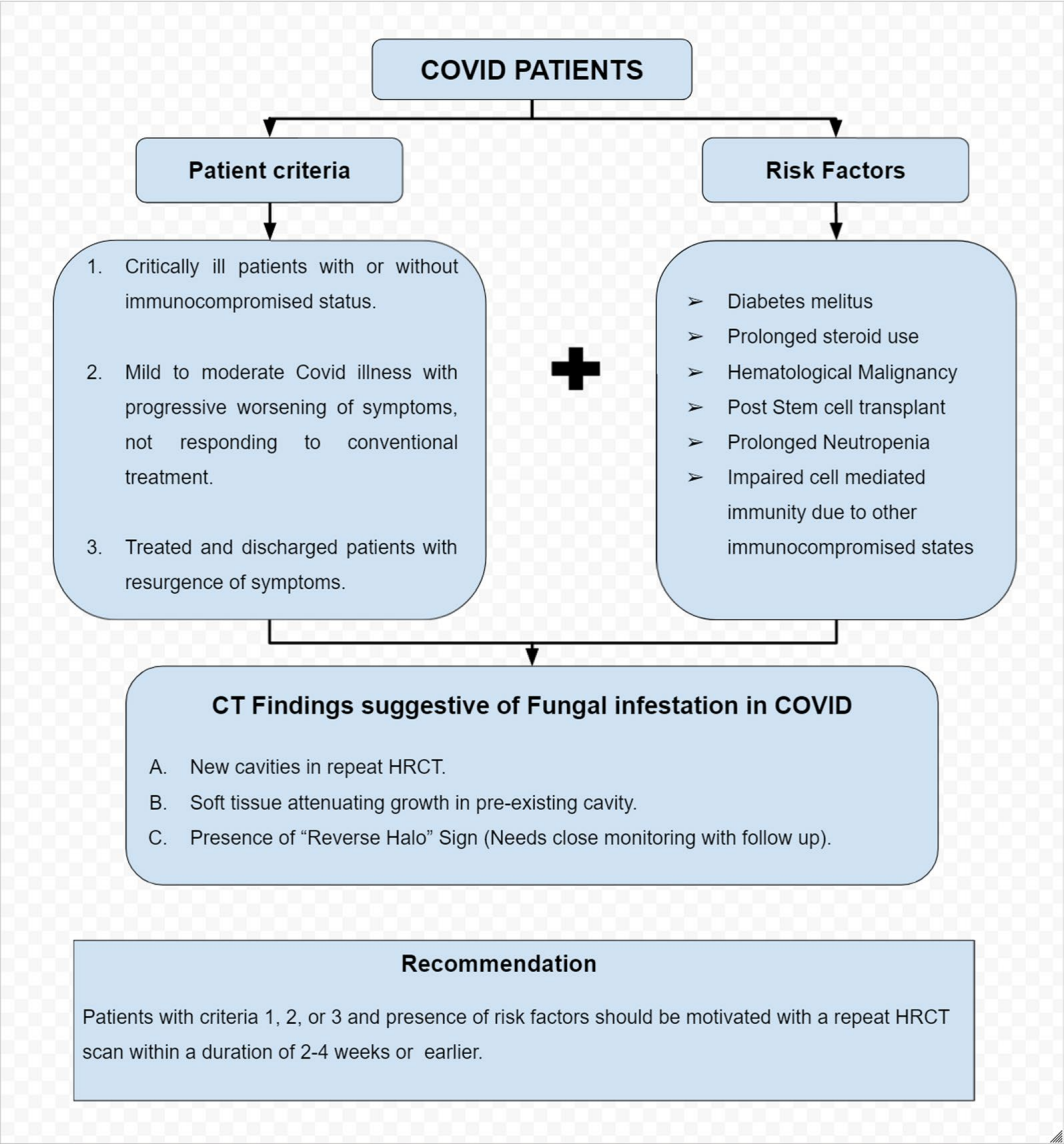
Recommended protocol for triaging the COVID patients with suspicious pulmonary fungal infection

A recommended protocol for detection of suspicious fungal infection in special categories of COVID patients is given in Fig. 7.

## Conclusion

Fungal co-infections linked to COVID-19 may be overlooked or often misdiagnosed. In India, an unfortunate combination of diabetes, widespread corticosteroid usage, and uncontrolled adverse effects of COVID-19 (cytokine storm, endotheliitis, lymphopenia) appears to be increasing the risk of fungal infections.

Most COVID patients undergo a HRCT thorax at some point. Hence, an alert skilful radiologist will pick up the characters of the residual lesion and association of this deadly virus early. In poorly managed diabetics or with other immunosuppressed conditions, invasive fungal infection (mucormycosis, invasive aspergillosis and invasive candidiasis) should be considered in the differential diagnosis of cavitary lung lesions. A protocol for HRCT scanning in COVID hospitals will go a long way in saving patients.

## Data Availability

The data were retrieved from our clinical and radiological database.

## Abbreviations

HRCT: High-resolution computed tomography
RT-PCR: Reverse transcriptase polymerase chain reaction
GGO: Ground-glass opacities
CKD: Chronic kidney disease
CRP: C-reactive protein
ICU: Intensive care unit
PTB: Pulmonary tuberculosis
